# Survival in gastric and esophageal cancers in the Nordic countries through a half century

**DOI:** 10.1002/cam4.5748

**Published:** 2023-02-27

**Authors:** Kari Hemminki, Filip Tichanek, Asta Försti, Otto Hemminki, Akseli Hemminki

**Affiliations:** ^1^ Biomedical Center, Faculty of Medicine Charles University Pilsen Czech Republic; ^2^ Division of Cancer Epidemiology German Cancer Research Center (DKFZ) Heidelberg Germany; ^3^ Institute of Pathological Physiology, Faculty of Medicine in Pilsen Charles University Pilsen Czech Republic; ^4^ Hopp Children's Cancer Center (KiTZ) Heidelberg Germany; ^5^ Division of Pediatric Neurooncology, German Cancer Research Center (DKFZ) German Cancer Consortium (DKTK) Heidelberg Germany; ^6^ Department of Urology Helsinki University Hospital and University of Helsinki Helsinki Finland; ^7^ Cancer Gene Therapy Group, Translational Immunology Research Program University of Helsinki Helsinki Finland; ^8^ Comprehensive Cancer Center Helsinki University Hospital Helsinki Finland

**Keywords:** mortality, relative survival, risk factors, stomach cancer, treatment

## Abstract

**Background:**

Gastric cancer (GC) and esophageal cancer (EC) are among the most fatal cancers and improving survival in them is a major clinical challenge. Nordic cancer data were recently released up to year 2019. These data are relevant for long‐term survival analysis as they originate from high‐quality national cancer registries from countries with practically free access to health care, thus documenting ‘real‐world’ experience for entire populations.

**Patients/Methods:**

Data were obtained for Danish (DK), Finnish (FI), Norwegian (NO), and Swedish (SE) patients from the NORDCAN database from years 1970 through 2019. Relative 1‐ and 5‐year survival were analyzed, and additionally the difference between 1‐ and 5‐year survival was calculated as a measure of trends between years 1 and 5 after diagnosis.

**Results:**

Relative 1‐year survival for Nordic men and women in GC was 30% in period 1970–74 and it increased close to 60%. Early 5‐year survival ranged between 10 and 15% and the last figures were over 30% for all women and NO men while survival for other men remain below 30%. Survival in EC was below that in GC, and it reached over 50% for 1‐year survival only for NO patients; 5‐year survival reached over 20% only for NO women. For both cancers, the difference between 1‐ and 5‐year survival increased with time. Survival was worst among old patients.

**Conclusion:**

GC and EC survival improved over the 50‐year period but the increase in 5‐year survival was entirely explained by gains in 1‐year survival, which improved at an accelerated pace in EC. The likely reasons for improvements are changes in diagnosis, treatment, and care. The challenges are to push survival past year 1 with attention to old patients. These cancers have a potential for primary prevention through the avoidance of risk factors.

## Graphical abstract

The study provides a unique survival analysis based on reliable diagnostic data over 50 years. It is extended to the most up‐to‐date year of 2019 showing positive development for both cancers. Figure: 1‐year survival in gastric cancer.

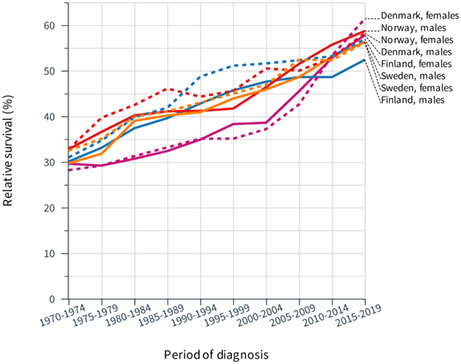

## 1 INTRODUCTION

Gastric (stomach) cancer (GC) has historically been the most common cancer in the world and in spite of its falling incidence, it remains among the most common cancers.^1^ International variation in incidence is large from high‐risk areas in most of Asia to low‐risk in Africa and Northern Europe.^2^, ^3^ The falling incidence trend in GC has been ascribed to declining rates in non‐cardia cancer due to *Helicobacter pylori* infections as a result of improved food preservation practices and household hygiene (called unplanned triumph of cancer prevention).^2^, ^3^ Other risk factors for GC include tobacco smoking, high‐salt diet, excess alcohol intake, obesity, family history, and low intake of fresh fruits and vegetables.^2^, ^3^, ^4^, ^5^ GC is often diagnosed at a late stage (III and IV) and localized tumors account for <10% of all, according to recent Danish and Norwegian data.^6^ Distant metastases are found most commonly in the liver; cardia tumors additionally metastasize to the lung, nervous system, and bone, whereas non‐cardia cancer more frequently metastasizes within the peritoneum.^7^ Treatment of GC involves surgical resection, supplemented with chemotherapy, and radiotherapy in localized disease, and in HER2‐positive cases targeted therapy; in metastatic disease, chemotherapy is offered.^8^ Anti‐angiogenic therapy and immunotherapy are novel options for advanced disease but it is unclear if they were used during the present study.^8^ Although survival rates in GC have been improving, the 5‐year relative survival rates have remained between 20 and 30% in developed countries by year 2010–2014.^9^, ^10^ Survival has reached over 60% in Japan and South Korea where population screening has been implemented.^11^

High‐incidence areas of esophageal cancer (EC) are East Asia and East Africa where squamous cell carcinoma is the most common histology; risk factors include tobacco smoking and alcohol but in the high‐risk areas other and unknown factors exist.^2^ In developed countries, adenocarcinoma has become the most common histology with risk factors of obesity and gastroesophageal reflux disease.^2^ Chronic reflux leads to metaplasia (Barrett esophagus) which may lead to adenocarcinoma.^12^ Intake of fruits and vegetables is thought to be protective against EC and family history is a risk factor.^2^, ^3^, ^4^, ^5^ Treatment of EC and GC is often centralized to specialist clinics as endoscopic and other surgical techniques with added chemo‐radiation are shared for the two cancers; chemotherapy is used in advanced disease.^8^, ^13^ EC metastasizes preferentially to the liver and lung.^7^ Survival has been poorer than in GC, 5‐year relative survival has ranged between 15 and 20% in developed countries by year 2010–2014.^9^, ^10^

Considering the international data on the dismal relative survival in GC and EC we wanted to assess survival in these cancers from Denmark (DK), Finland (FI), Norway (NO), and Sweden (SE) where survival data are available at the national level from 1970 to 2019. Health care access has traditionally been available with minimal costs to the population at large allowing these countries to stage the real‐world experience in cancer outcomes. Health care resources depend on the countries' economic prosperity and the share used for health care. In 2000, health care expenditure per capita was $2496 (8.8% of GNP) in DK, $1723 (7.1%) in FI, $2949 (7.7%) in NO, and $2173 (7.3%) in SE. We show data on expenditure per capita and its share of GNP for years 1970, 1990, and 2010 in Table 1. Life expectancy increased between 1970 and 2019 in FI by 11.6 years and in other countries by 8 years. We show data on the development of 1‐year and 5‐year survival between periods 1970–4 to 2015–9, and also calculate survival difference between years 1 and 5 as an indication of improvement in survival between years 1 and 5. These data should help interpret survival improvements in these cancers.

**TABLE 1:** Health care expenditure per capita and its share of the gross national product (GNP in US$) in the Nordic countries (adjusted for purchase power parity) (www.macrotrends.net).

Country	1970		1990		2010	
	Expenditure/capita	% of GNP	Expenditure/capita	% of GNP	Expenditure/capita	% of GNP
Denmark	216	5.9	1424	8.3	4464	11.1
Finland	163	5.7	1292	8.0	3256	8.9
Norway	131	4.5	1365	7.8	5388	9.4
Sweden	270	7.1	1492	8.8	3758	9.6

## 2 MATERIALS AND METHODS

The data used originate from the NORDCAN database which is a compilation of data from the Nordic cancer registries as described.^14^, ^15^ These registries are presented in detail by Pukkala and coworkers.^16^ The database was accessed at the IARC website (https://nordcan.iarc.fr/en/database#bloc2). NORDCAN uses International Classification of Diseases version 10 codes (C16 for GC and C15 for EC) for which the codes from earlier versions were translated.

Survival data were available from 1970 through 2019 and the analysis was based on the cohort survival method for periods from 1970 to 2014, and a hybrid analysis combining period and cohort survival in the last period 2015–2019, as detailed.^14^, ^15^ Age‐standardized relative survival was estimated using the Pohar Perme estimator.^17^ Age‐standardization was performed by weighting individual observations using external weights as defined at the IARC website. National general population life‐tables stratified by sex, year, and age were used in the calculation of expected survival. Death certificate‐only cases were not included. Patients 90 years or older were excluded. Groups were analyzed if the minimum of 30 patients were alive at the start and with minimum of 3 patients in any one of age‐groups used for weights. Age‐specific survival was assessed in an earlier version of NORDCAN in which follow‐up started in 1967 and ended in 2016 (https://www‐dep.iarc.fr/NORDCAN/english/frame.asp). Incidence data were plotted from the NORDCAN site using age standardization to the world population. In graphic presentation of incidence data, smoothing was used.

We calculated also a difference in survival percent between year 1 and year 5 as a measure of how well survival is maintained between years 1 and 5.^18^, ^19^ When comparing survival trends, a decreasing difference between years 1 and 5 indicates improving survival between years 1 and 5 after diagnosis.

## 3 RESULTS

Survival trends should be viewed with knowledge on the incidence trends in particular cancers. In Figure S1 incidence rates for GC are plotted for the Nordic populations. FI men had the highest incidence of 34/100,000 in 1970 and SE and DK men had the lowest incidence of 20/100,000, only slightly higher than the FI female rate. In the 50‐year period, all rates markedly declined, with the exception of the DK male rate which modestly increased in the last 10 years. For EC, DK male rate increased more than twofold to a level of 7/100,000, two times higher than the other male rates, which had been relatively stable through the 50‐year period (Figure S2). NO and SE female rates had been stable at 1/100,000 to which level the FI female decreased by year 2000 and crossed the increasing DK female rate after 1990.

**FIGURE 1:**
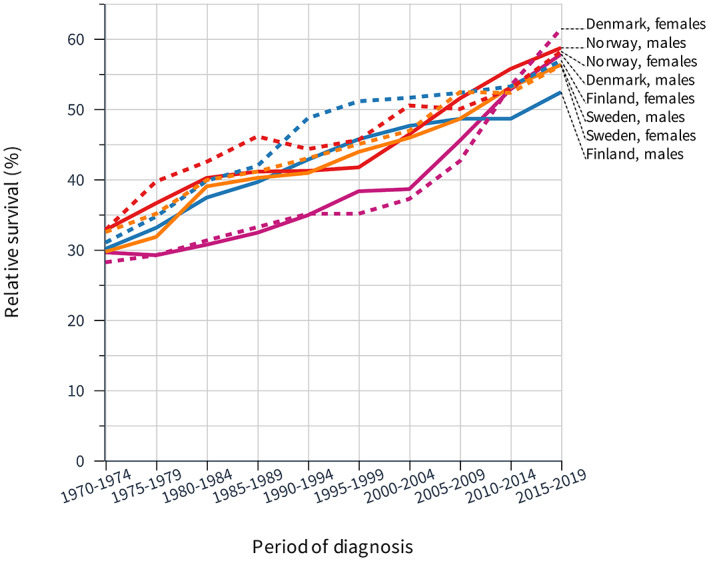
Relative 1‐year survival in gastric cancer among Nordic men and women between 1970 and 2019.

Relative 1‐year survival for Nordic men and women in GC is shown in Figure 1 for the 50‐year period. Survival was 30% in the period 1970–4 and it increased close to 60% for men and women. Initially, the survival improved slowly for DK men and women but after year 2000 there was a steep increase and catching up. Survival for FI men lagged behind in the last 10‐year period.

The underlying survival data are shown in Table 2, indicating significant improvements (non‐overlapping 95% CIs) in survival, compared to the previous 5‐year period (indicated by an asterix). Note that 10‐year intervals were compared in this table in order to reduce random variation. For FI men the final survival value of 50.0% was significantly below that of the other countries.

**TABLE 2:** Relative 1‐ and 5‐year survival (% with 95% CI) and their difference (Diff) in gastric cancer in the Nordic countries.

Stomach, 1 year, Male									Stomach, 1 year, Female							
Label	Denmark		Finland		Norway		Sweden		Denmark		Finland		Norway		Sweden	
1970–1979	29.2 [28.0–30.5]		32.0 [30.8–33.2]		34.6 [33.4–35.9]		30.6 [29.6–31.5]		28.4 [26.9–30.0]		32.8 [31.5–34.1]		36.0 [34.4–37.6]		33.5 [32.3–34.7]	
1980–1989	31.5 [30.1–32.9]		38.8 [37.5–40.1]*		40.5 [39.1–41.9*]		39.5 [38.4–40.7]*		31.5 [29.7–33.4]		40.5 [39.1–42.0]		43.7 [41.9–45.5]*		40.2 [38.8–41.6]*	
1990–1999	36.5 [34.8–38.3]*		44.1 [42.6–45.6]*		41.3 [39.7–43.0]		42.3 [41.0–43.6]*		34.8 [32.7–37.0]		49.1 [47.4–50.8]*		44.7 [42.5–46.9]		43.4 [41.8–45.0]*	
2000–2009	42.3 [40.5–44.0]*		48.1 [46.5–49.8]*		48.6 [46.6–50.4]*		46.6 [45.2–48.0]*		39.6 [37.2–42.0]*		51.2 [49.3–53.1]		49.8 [47.4–52.1]*		48.6 [46.8–50.4]*	
2010–2019	55.0 [53.3–56.7]*		50.0 [48.2–51.8]		56.7 [54.6–58.6*]		54.0 [52.5–55.5]*		56.9 [54.5–59.2]*		54.3 [52.2–56.4]		54.2 [51.5–56.8]		53.1 [51.2–55.0]*	

Stomach, 5 years, Male		Diff		Diff		Diff		Diff	Stomach, 5 years, Female	Diff		Diff		Diff		Diff
1970–1979	11.0 [10.0–11.9]	18.2	12.9 [11.9–13.8]	19.1	15.9 [14.9–17.0]	18.7	12.9 [12.2–13.7]	17.7	11.8 [10.6–13.1]	16.6	13. 2 [12.2–14.3]	19.6	14.5 [13.2–15.8]	21.5	14.5 [13.5–15.5]	19
1980–1989	12.5 [11.4–13.7]	19	19.0 [17.8–20.2]*	19.8	19.4 [18.1–20.7]*	21.1	17.6 [16.7–18.6]*	21.9	14.3 [12.8–15.9]	17.2	19.6 [18.4–20.9]*	20.9	22.5 [20.8–24.2]*	21.2	19.9 [18.7–21.2]*	20.3
1990–1999	13.5 [12.2–14.9]	23	23.0 [21.5–24.4]*	21.1	18.9 [17.5–20.4]	22.4	19.7 [18.6–20.9]	22.6	15.6 [13.9–17.5]	19.2	28.0 [26.4–29.7]*	21.1	23.6 [21.6–25.6]	21.1	21.5 [20.0–22.9]	21.9
2000–2009	15.7 [14.3–17.1]	26.6	24.0 [22.4–25.6]	24.1	22.7 [21.0–24.5]*	25.9	20.8 [19.5–22.1]	25.8	16.6 [14.7–18.6]	23	28.6 [26.7–30.5]	22.5	24.8 [22.6–27.1]	25	25.2 [23.6–26.9]*	23.4
2010–2019	25.9 [24.1–27.7]*	29.1	24.6 [22.7–26.5]	25.4	28.9 [26.7–31.1]*	27.8	26.0 [24.3–27.6]*	28	28.5 [25.8–31.3]*	28.4	29.6 [27.3–32.0]	24.7	29.0 [26.1–31.9]	25.2	29.8 [27.8–31.8] *	23.3

*Note*: Diff = difference between 1‐ and 5 year survival in % units.

* The 95% CIs were non‐overlapping for survival between this and the previous period.

**FIGURE 2:**
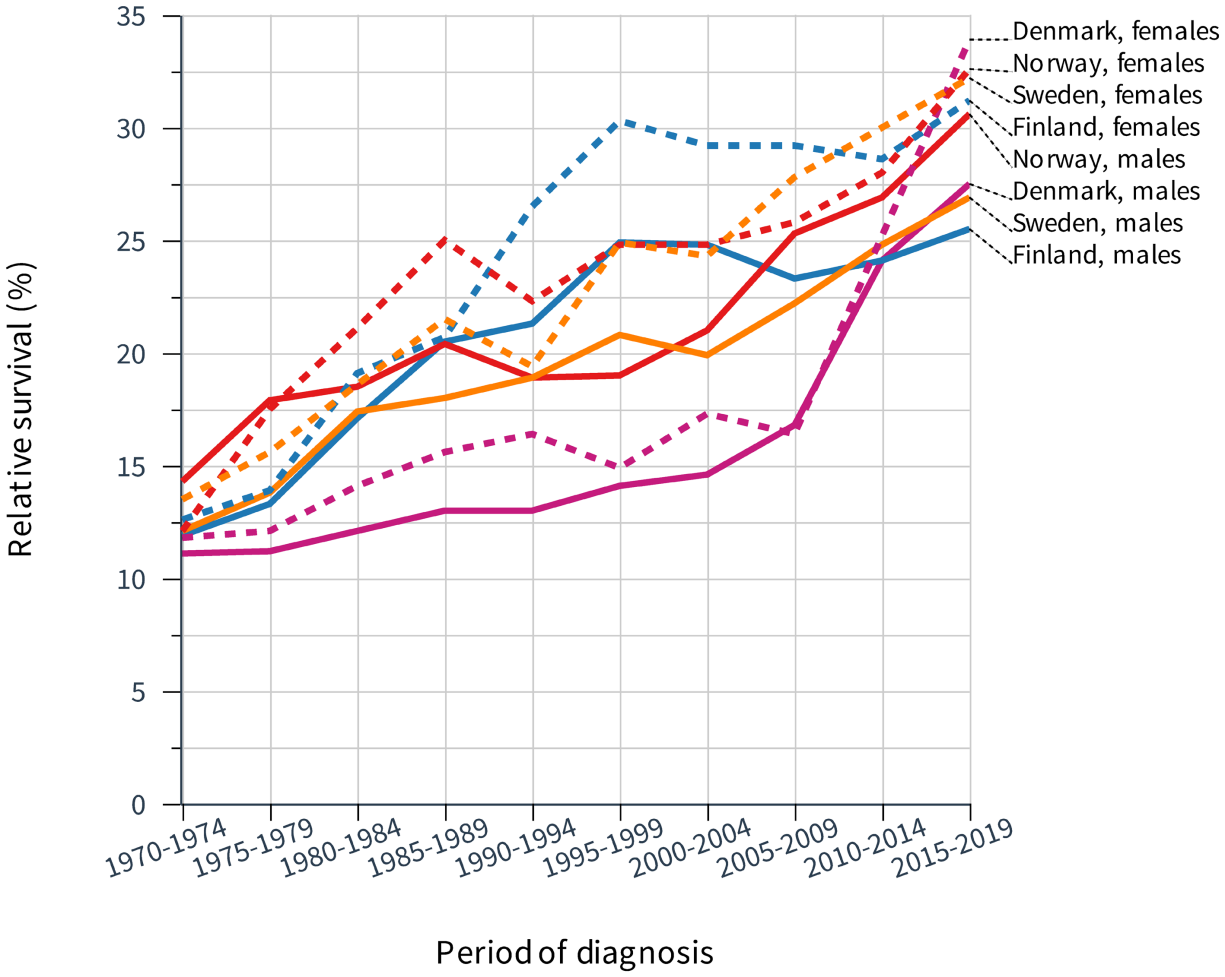
Relative 5‐year survival in gastric cancer among Nordic men and women between 1970 and 2019.

GC 5‐year survival is plotted in Figure 2 and it shows much larger dispersion of data compared to 1‐year survival. The first survival figures range between 10% and 15% and the last figures are over 30% for all women and NO men while survival for other men remain below 30%. The actual survival figures are shown in Table 2. It shows additionally the difference between year 1 and 5 survival (‘Diff’) which increased with time and the increase was highest, over 10% units, for DK men and women.

**FIGURE 3:**
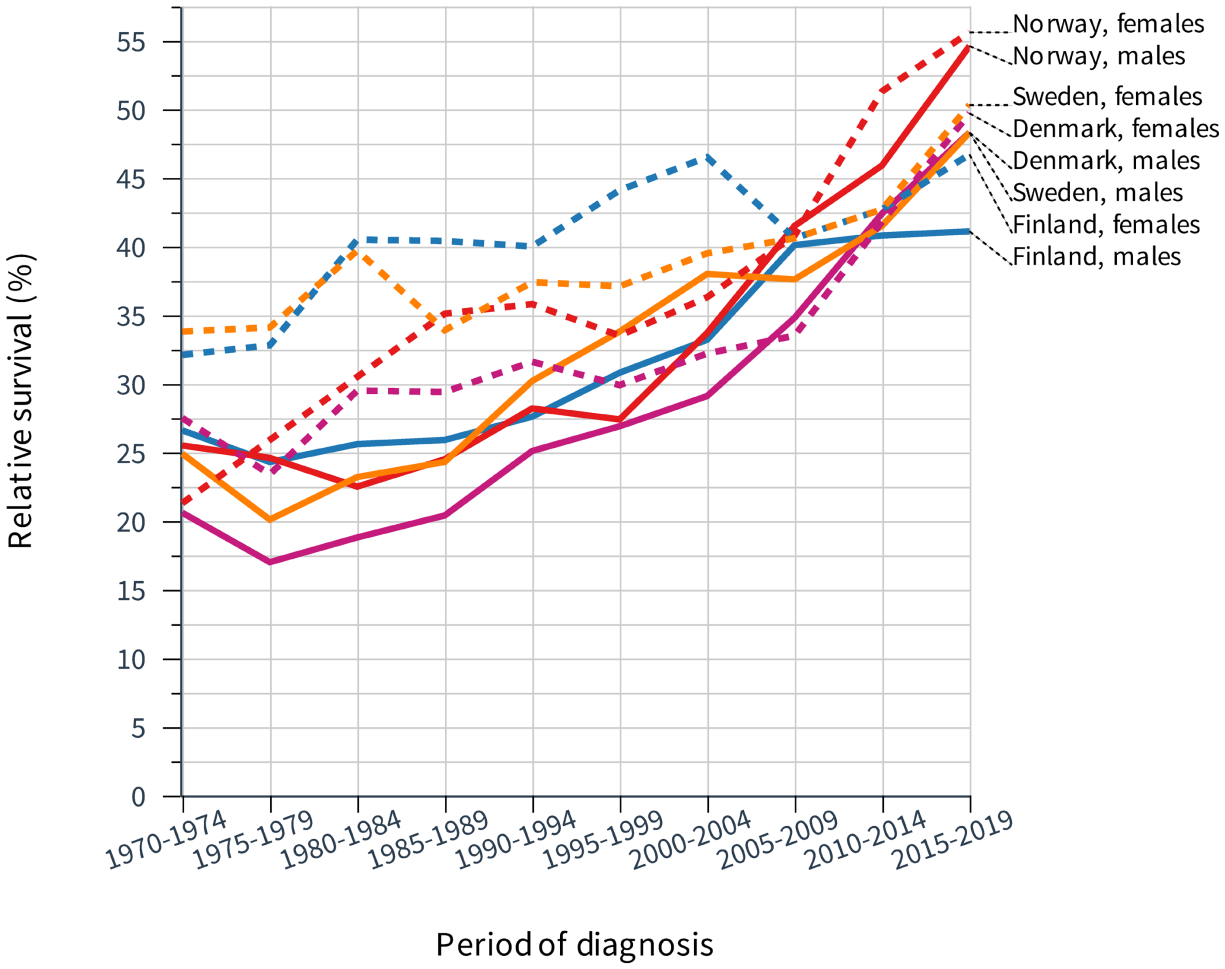
Relative 1‐year survival in esophageal cancer among Nordic men and women between 1970 and 2019.

Relative 1‐year survival in EC is shown in Figure 3. Male survival was initially about 25% (DK 20%) and it hardly improved until year 2000 but thereafter a steep increase followed. Female survival modestly improved throughout the follow‐up time but at the end, the male and female survival curves reached each other. Table 3 witnesses the superior NO performance for men and women and the final survival figures (49.8% for men and 53.9% for women) were significantly better than those of many Nordic colleagues. FI performed poorly during the final periods and the last survival figures were significantly lower than those of many Nordic colleagues.

**TABLE 3:** Relative 1‐ and 5‐year survival (% with 95% CI) and their difference (Diff) in esophageal cancer in the Nordic countries.

Esophagus, 1 year, Male									Esophagus, 1 year, Female							
Label	Denmark		Finland		Norway		Sweden		Denmark		Finland		Norway		Sweden	
1970–1979	18.7 [16.2–21.4]		24.9 [22.0–27.8]		24.3 [21.2–27.5]		22.8 [20.7–24.8]		24.1 [20.0–28.4]		33.1 [30.0–36.3]		22.1 [16.7–27.9]		34.4 [30.6–38.3]	
1980–1989	19.5 [17.3–21.7]		25.5 [22.6–28.5]		23.8 [20.9–26.9]		23.6 [21.7–25.6]		30.2 [26.4–34.1]		39.8 [36.3–43.4]		34.8 [28.6–41.1]*		35.1 [31.4–38.8]	
1990–1999	25.9 [24.0–27.8]*		29.6 [26.9–32.4]		27.5 [24.8–30.3]		32.0 [30.0–34.0]*		30.3 [27.0–33.6]		42.3 [38.4–46.3]		33.8 [28.7–39.0]		37.6 [34.2–41.1]	
2000–2009	31.7 [29.9–33.5]*		36.9 [34.3–39.4]*		37.0 [34.3–39.7]*		37.7 [35.8–39.5]*		32.1 [29.3–35.0]		41.9 [37.9–45.8]		37.3 [32.7–41.8]		39.2 [36.0–42.4]	
2010–2019	46.4 [44.7–48.1]*		41.5 [39.2–43.7]		49.8 [47.5–52.2]*		43.7 [41.8–45.6]*		46.6 [43.6–49.5]*		42.5 [38.3–46.5]		53.9 [49.5–58.0]*		44.9 [41.8–48.1]	

Esophagus, 5 years, male		Diff		Diff		Diff		Diff	Esophagus, 5 years, female	Diff		Diff		Diff		Diff
1970–1979	2.8 [1.8–4.3]	15.9	4.4 [3.0–6.2]	20.5	3.0 [1.8–4.8]	21.3	5.5 [4.4–6.9]	17.3	6.3 [4.1–9.0]	17.8	8.2 [6.4–10.3]	24.9	5.5 [2.9–9.5]	16.6	8.8 [6.5–11.5]	25.6
1980–1989	3.4 [2.3–4.6]	16.1	7.3 [5.5–9.4]	18.2	4.2 [2.8–6.0]	19.6	5.8 [4.7–7.1]	17.8	8.7 [6.4–11.5]	21.5	11.9 [9.4–14.6]	27.9	10.4 [6.7–15.0]	24.4	11.2 [8.6–14.1]	23.9
1990–1999	4.8 [3.8–5.9]	21.1	7.3 [5.7–9.2]	22.3	6.4 [4.8–8.2]	21.1	9.8 [8.5–11.3]*	22.2	6.1 [4.4–8.1]	24.2	15.8 [12.7–19.3]	26.5	9. 8 [6.6–13.8]	24	13.7 [11.2–16.5]	23.9
2000–2009	8.5 [7.4–9.7]*	23.2	11.6 [9.9–13.5]*	25.3	9.8 [8.1–11.6]	27.2	11.9 [10.6–13.3]	25.8	11.8 [9.8–14.0]*	20.3	16.7 [13.7–20.0]	25.2	11.5 [8.6–15.0]	25.8	13.3 [11.0–15.8]	25.9
2010–2019	17.2 [15.6–18.8]*	29.2	14.9 [13.0–16.9]	26.6	21.3 [19.0–23.7]*	28.5	15.0 [13.3–16.7]	28.7	17.6 [15.0–20.5]*	29	17.7 [14.0–21.7]	24.8	27.7 [22.9–32.7]*	26.2	18.9 [15.9–22.1]*	26

*Note*: Diff = difference between 1‐ and 5‐year survival in % units.

* The 95% CIs were non‐overlapping for survival between this and the previous period.

**FIGURE 4:**
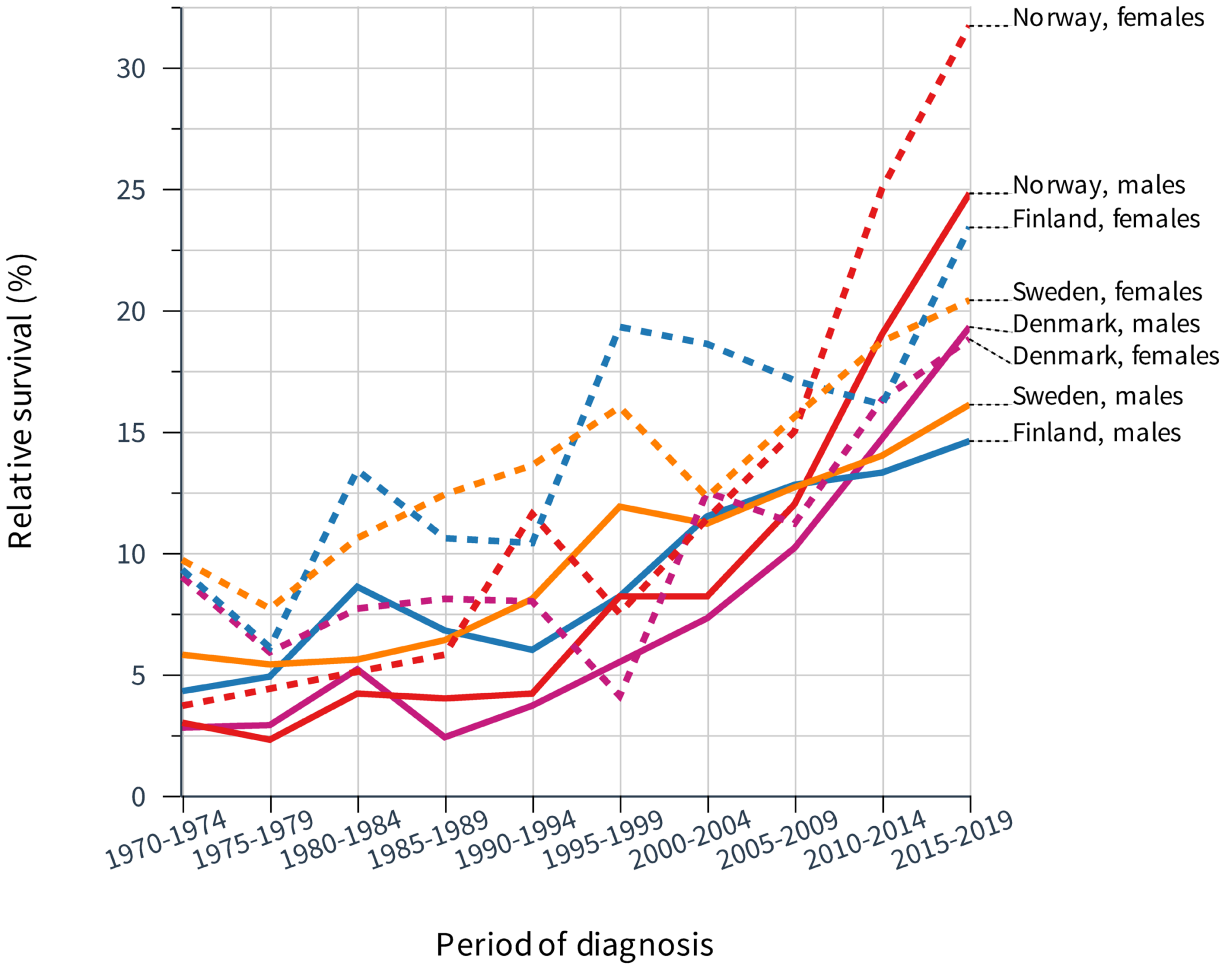
Relative 5‐year survival in esophageal cancer among Nordic men and women between 1970 and 2019.

Differences in EC 5‐year survival plots widened over time (Figure 4). NO men and women started at <5% survival and ended up at 25% and over 30%, respectively. FI and SE men started at 5% and ended up at 15%. Table 3 witnesses the superior NO performance also for 5‐year survival which for men and women were significantly better than those of any Nordic colleagues. The difference between 1‐ and 5‐year survival increased with time and it reached 25%–29% units in the final period.

Age‐specific survival data were generated from an earlier version of NORDCAN and results for GC for SE are shown in Figure S3. Survival in age group 80–89 years was clearly worse than in younger age groups and the difference appeared to widen with time; among 80–89 years old, 13% of SE men and women were alive in 5 years in 2012–16. The data for the other Nordic countries were similar; in 2012–16, 5‐year survival among 80–89 years old in DK was 12/7% (men/women), in FI 15/18%, and in NO 17/19%.

Age‐specific data for EC in SE are shown in Figure S4. Survival in age group 80–89 years did not improve over time, and 5‐year survival in this age group remained at 4/5% (men/women) in SE. The situation among the 80–89 years old was no better in the other Nordic countries; in 2012–16, 5‐year survival in DK was 4/3%, in FI 3/7%, and in NO 3/5%.

## 4 DISCUSSION

Novel findings of the present study include demonstration of recent positive development in 1‐ and 5‐year survival for GC and EC which for GC has been relatively uniform over the 50‐year period but for EC most gains were achieved after year 2000, particularly for men. Survival in female GC has been better than in male cancer, and the differences remained particularly in 5‐year survival. For EC, female survival was far better than male survival until about 2004 but in the last 15 years, males have been able to narrow the gap. An important and perhaps surprising observation was the favorable 5‐year survival for GC and EC was entirely due to the vast gains in 1‐year survival. The difference between years 1 and 5 survival increased with time suggesting that novel diagnostics, treatment, and care were helping increasing numbers of patients survive past year 1 but succumb before reaching 5 years. This is due to the strong increase in 1‐year survival and weaker development in survival between years 1 and 5. A recent survival study from years 2012 to 2014, including DK and NO, appears to support our assumptions.^6^ Stages I and II (or localized) GC and EC, accounting for about 20% of all, maintained relatively good survival up to year 3 (no 5‐year data were available) while stage IV (distant) had dropped to <5%. Stage III data were available only for DK, and survival between years 1 and 3 was more than 50%.

As survival is dependent on incidence, survival data should be interpreted with caution for cancers for which incidence has changed.^20^ For GC, the incidence has markedly decreased in all countries, and mostly for FI men and women (Figure S1). However for EC, the country‐specific trends have differed, DK with increasing, FI with decreasing, and NO and SE with stable trends (Figure S1). The reasons may be related to smoking, alcohol drinking, and, particularly, their interactions.^21^ While smoking levels have generally decreased in Nordic countries, smoking prevalence has remained high in DK.^22^, ^23^ Consumption of alcohol has also been highest in DK which may explain the high DK incidence trends for EC.^23^ It is, however, not well known how these risk factors might be related to survival trends, and the recent DK survival in EC was essentially at the level of FI and SE.

The difference between years 1 and 5 survival has been recently reported in several cancers from FI and SE. In both countries, survival difference decreased with time for bladder cancer and for renal cell cancer in FI only.^19^, ^24^ For colorectal cancer, the difference narrowed over time for rectal cancer but for colon cancer, it remained constant.^18^ We assume that survival improvement between years 1 and 5 is likely to indicate earlier diagnosis and true treatment benefits in at least locally advanced tumors, as in rectal cancer surgery and radiotherapy have improved late survival.^18^ The time‐dependent increase in survival difference between years 1 and 5 in GC and EC is probably the consequence of organizational and therapeutic changes extending survival past year 1.^9^ The Swedish National Register for Esophageal and Gastric Cancer witnesses a vast increase in presentation of patients to multidisciplinary teams, shorter times from referral to these teams and a centralization of treating hospitals in a 10‐year period.^25^ The changes in treatment were increase in minimally invasive surgery, decline in resection rates (excluding endoscopic resections) and an increased application of preoperative treatment with chemotherapy for GC and with chemo‐radiotherapy for EC.^25^ The current treatment guidelines for local GC include preoperative and postoperative chemotherapy.^26^ For locally advanced EC the guidelines recommend pre‐ and perioperative treatment using chemotherapy and/or chemo‐radiotherapy.^27^ For metastatic GC and EC immunotherapy with or without chemotherapy is recommended.^26^, ^27^

Survival differed by age groups and GC patients diagnosed after 80 years survived worse than younger patients while those diagnosed before age 70 years showed only minor differences. For EC age‐specific differences were larger than for GC and patients diagnosed at age over 80 years did not benefit from the overall survival improvement; their 5‐year survival remained at a miserable 5%. Poor survival among old patients have been ascribed to lower treatment in general and limitations because of comorbidities.^9^ Comorbidities influence survival in all EC patients.^28^

The health care organization in the Nordic countries has been basically similar but economic resources influence investments in staff and in diagnostic and treatment facilities. For GC, survival in DK was lagging behind until about year 2000 when a fast improvement started, in identical tempo in men and women. By the end of follow‐up, DK caught up with the other countries. Another feature of GC survival was that improvements in FI slowed down at around 2000. For EC, the experience in DK and FI resembled that for GC. NO excelled for EC by starting with modest survival rates but ending up clearly on top among men and women. Prosperity in NO has increased throughout the study period, starting with the lowest Nordic GNP in 1970 and clearly passed the other countries by 2010 (Table 1). By 2019 the NO expenditure ended up at 40% higher than SE and DK and 50% higher than FI in 2019. In terms of purchasing power parity (PPP), the differences are not as large because of higher salary and cost level in NO. During the 1980 s the proportion of health care expenditure of GNP dropped in SE and particularly in DK. FI experienced the worst economic depression since the Second World War in the 1990 s when GNP slumped and, additionally, the health care share of GNP was reduced. In 2008/2009 all countries went into temporary economic depression, which was weakest in NO. Even though we have no way to causally link the macroeconomic changes to output of health care for GC and EC, one can assume that the superior NO survival achievements may have benefitted from the solid economic support. The initially sluggish survival development in DK, and the sagging FI outcomes into year 2000 followed the economic squeeze. DK was the first Nordic country to set up a national cancer plan in year 2000; this ensured funding for cancer care and brought about administrative changes for accelerated cancer care pathways.^29^ Table 1 shows that DK uses the highest share of GNP in health care compared to the other Nordic countries. NO and SE drafted their cancer plans later but for FI it is yet to be accomplished.^30^

The main limitations of the study are lacking detailed pathological and clinical data, including histology, stage, and treatment. The strengths are long‐term follow‐up at the national level and availability of high‐quality data from the cancer registries, which have a history of close collaboration. Reliable survival data spanning a half century are available nowhere outside the Nordic countries, and follow‐up to the end of 2019 guarantees the most up‐to‐date survival results.

In conclusion, the present follow‐up of GC and EC to the end of 2019 witnessed a positive development in 1‐year survival which improved at an accelerated pace in EC. The likely reasons are changes in diagnostic, treatment, and care practice. The challenges are to push survival past year 1 and to enroll old patients who have benefitted so far least. It has been estimated that 50%–75% of these cancers are associated with environmental risk factors suggesting a large potential for primary prevention.^31^, ^32^ Population screening in the Japanese‐Korean style has not been considered feasible in the low‐incidence countries but *H. pylori* infections and Barrett esophagus can be treated; aspirin and nonsteroidal anti‐inflammatory drugs may reduce risks but have side effects.^1^ Biomarker development may allow detection of early lesions in the future. Immunotherapy has improved outcomes in some cancers and now the first positive results were reported for nivolumab, a PD‐1 inhibitor, in combination with chemotherapy in metastatic GC and EC.^33^

## AUTHOR CONTRIBUTIONS

**Kari Hemminki:** Conceptualization (lead); funding acquisition (equal); project administration (lead); supervision (lead); writing – original draft (lead). **Filip Tichanek:** Methodology (lead); software (equal); visualization (equal). **Asta Försti:** Investigation (equal); validation (equal); writing – review and editing (equal). **Otto Hemminki:** Investigation (equal); validation (equal); writing – review and editing (equal). **Akseli Hemminki:** Investigation (equal); validation (equal); writing – review and editing (equal).

## FUNDING INFORMATION

Supported by the European Union's Horizon 2020 research and innovation program, grant No 856620, Jane and Aatos Erkko Foundation, Sigrid Juselius Foundation, Finnish Cancer Organizations, University of Helsinki, Helsinki University Central Hospital, Novo Nordisk Foundation, Päivikki and Sakari Sohlberg Foundation.

## CONFLICT OF INTEREST STATEMENT

A.H. is shareholder in Targovax ASA. A.H. is employee and shareholder in TILT Biotherapeutics Ltd. Other authors declared no conflict of interest.

## ETHICS STATEMENT

Publically available data without individual identifies pose no ethical issues.

## Supporting information


Figures S1–S4.

## Data Availability

Publicly available data at https://nordcan.iarc.fr/en/database#bloc2.
