# Supplementary material for: Survival in gastric and esophageal cancers in the Nordic countries through a half century
Source: Cancer Med. 2023 Feb 27;12(9):10212–21. doi: 10.1002/cam4.5748 (PMC10225220; doi:10.1002/cam4.5748)
Supplement: Supplementary file 1 — Figures S1–S4. [file CAM4-12-10212-s001.pptx]

## Slide 1
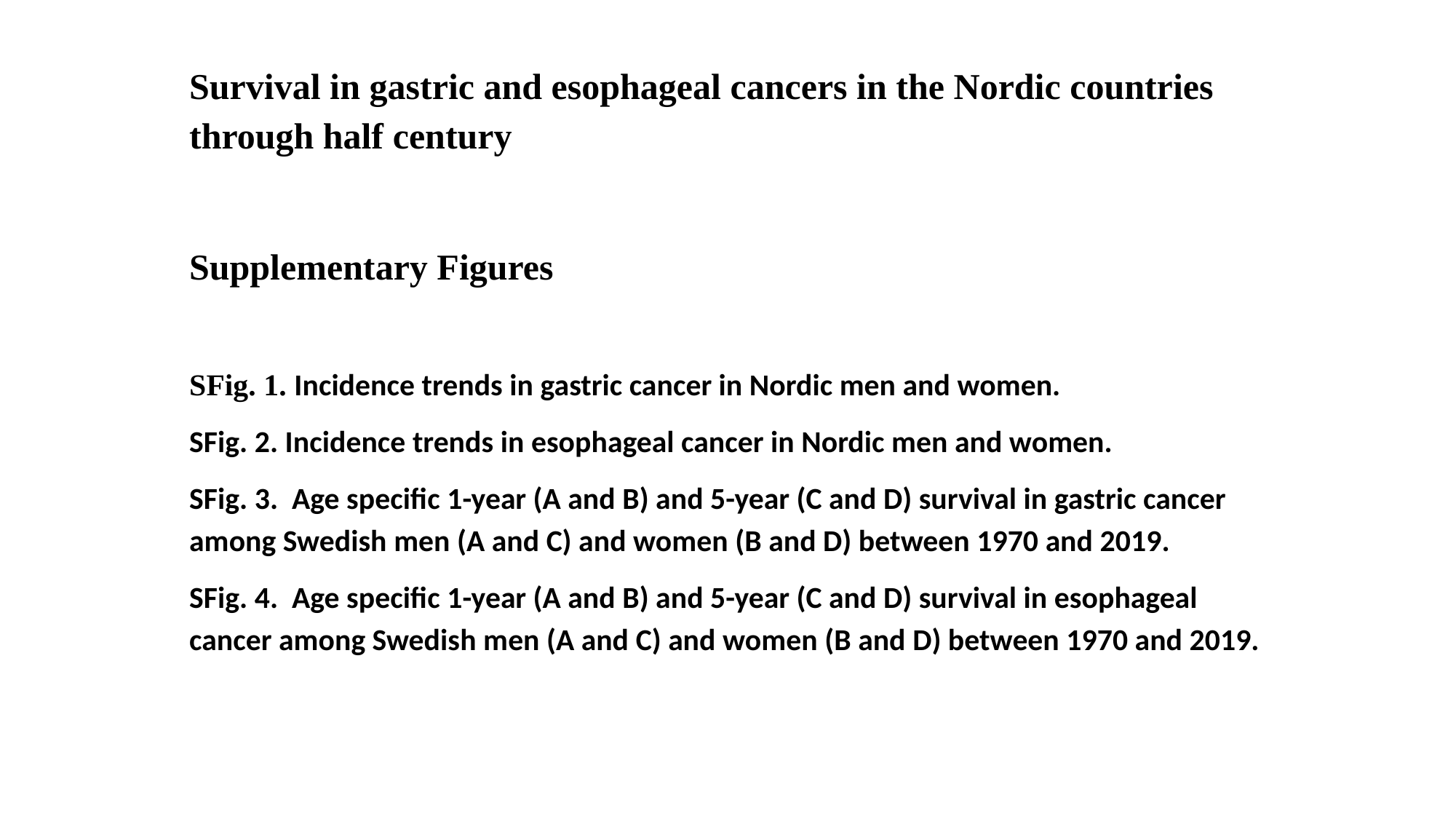

Survival in gastric and esophageal cancers in the Nordic countries through half century
Supplementary Figures
SFig. 1. Incidence trends in gastric cancer in Nordic men and women.
SFig. 2. Incidence trends in esophageal cancer in Nordic men and women.
SFig. 3. Age specific 1-year (A and B) and 5-year (C and D) survival in gastric cancer among Swedish men (A and C) and women (B and D) between 1970 and 2019.
SFig. 4. Age specific 1-year (A and B) and 5-year (C and D) survival in esophageal cancer among Swedish men (A and C) and women (B and D) between 1970 and 2019.

## Slide 2
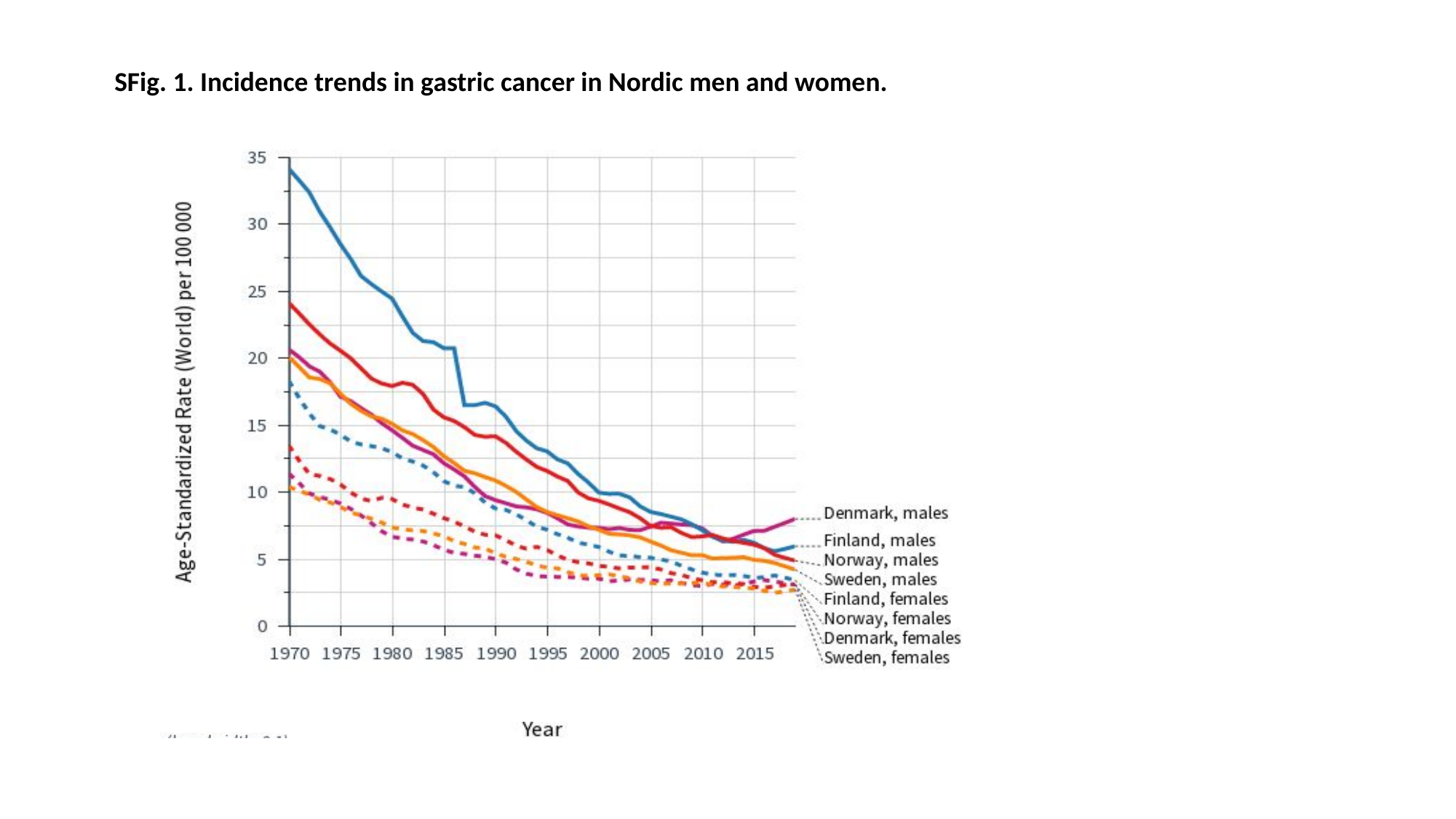

SFig. 1. Incidence trends in gastric cancer in Nordic men and women.

## Slide 3
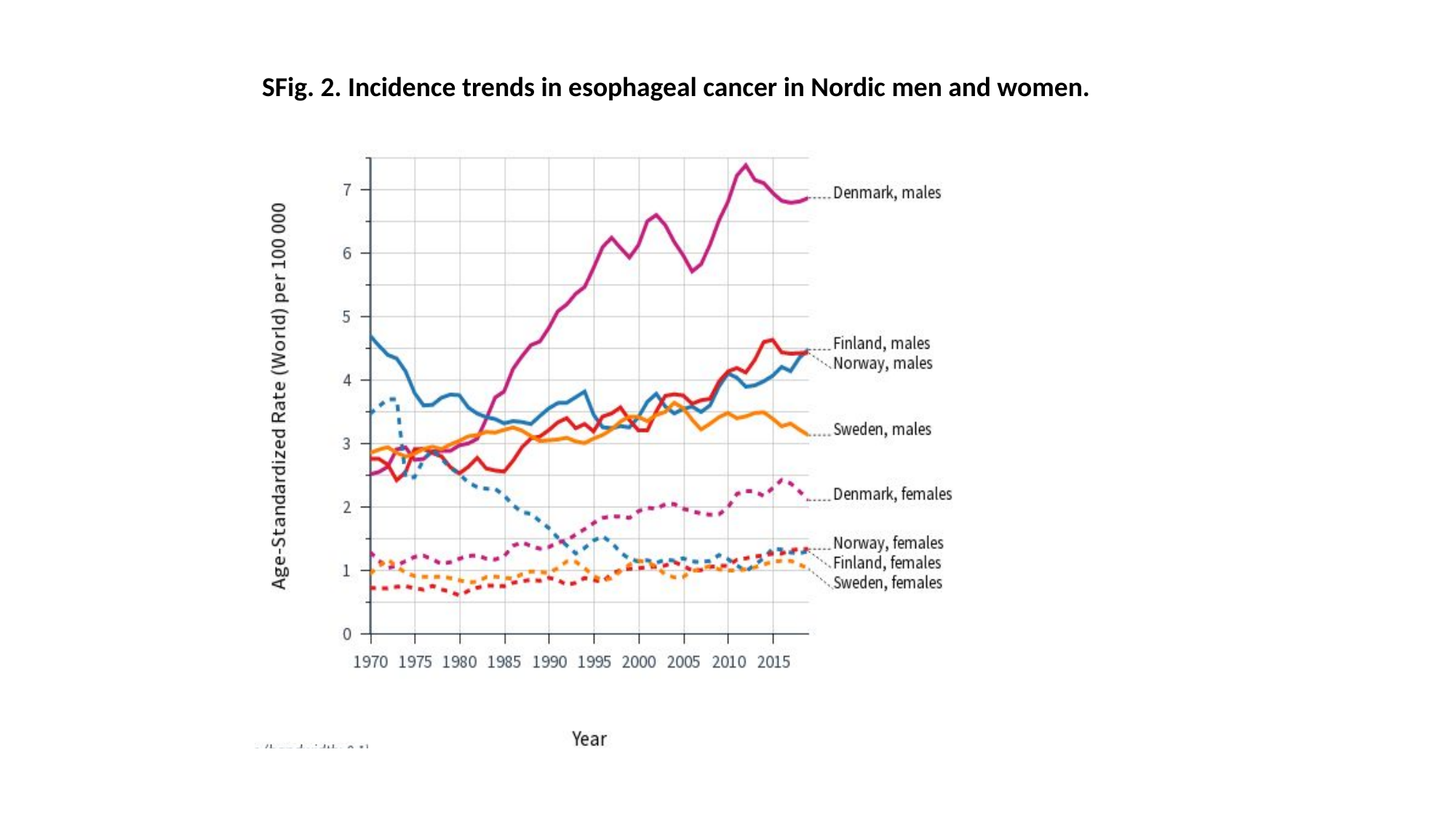

SFig. 2. Incidence trends in esophageal cancer in Nordic men and women.

## Slide 4
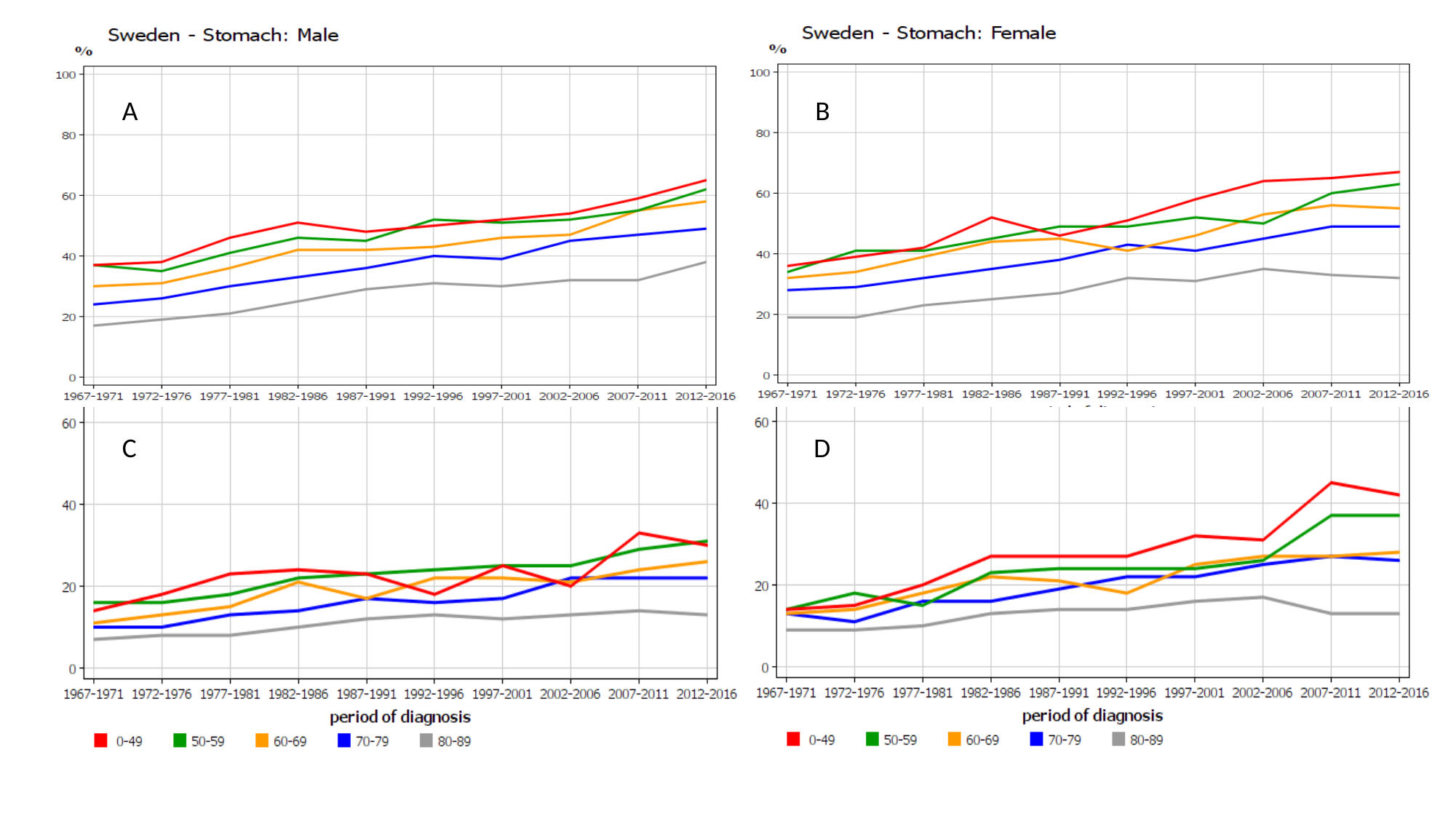

A
B
C
D

## Slide 5
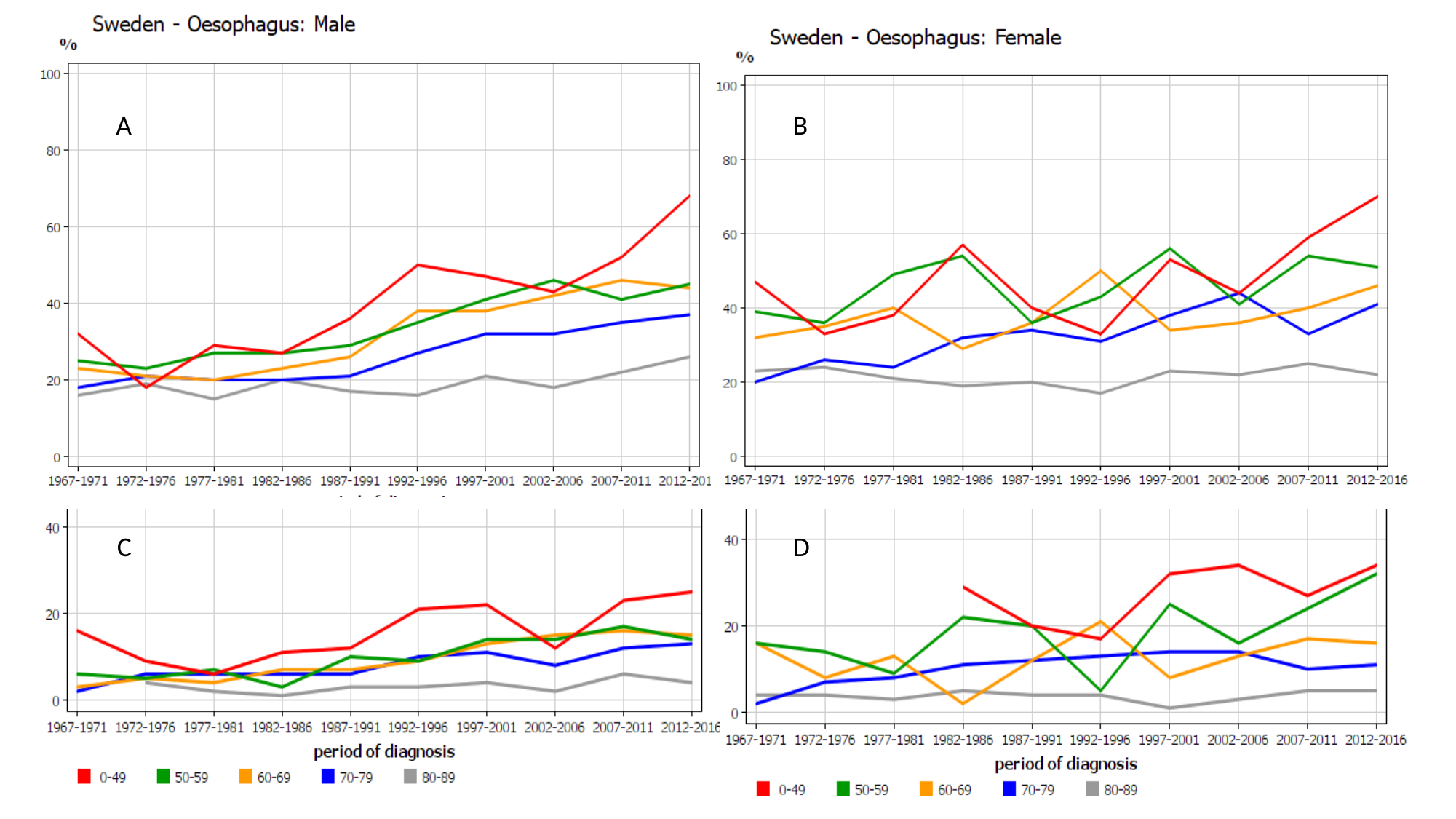

A
B
C
D
